# Wilder rangelands as a natural climate opportunity: Linking climate action to biodiversity conservation and social transformation

**DOI:** 10.1007/s13280-023-01976-4

**Published:** 2024-01-31

**Authors:** Lavhelesani D. Simba, Mariska te Beest, Heidi-Jayne Hawkins, Keith W. Larson, Anthony R. Palmer, Camilla Sandström, Kathleen G. Smart, Graham I. H. Kerley, Joris P. G. M. Cromsigt

**Affiliations:** 1 Centre for African Conservation Ecology, Nelson Mandela University, P.O. Box 77000, Gqeberha, 6031 South Africa; 2 Copernicus Institute of Sustainable Development, Utrecht University, Utrecht, The Netherlands; 3 South African Environmental Observation Network (SAEON), Grasslands, Forests and Wetlands Node, Pietermaritzburg, South Africa; 4 Conservation International, Forrest House, Belmont Park, Rondebosch, Cape Town, 7700 South Africa; 5 Department of Biological Sciences, University of Cape Town, Rondebosch, Private Bag X1, Cape Town, 7701 South Africa; 6 Department of Ecology and Environmental Science, Climate Impacts Research Centre, Umeå University, 901 87 Umeå, Sweden; 7 Institute for Water Research, Rhodes University, Makhanda, 6139 South Africa; 8 Department of Political Science, Umeå University, 90187 Umeå, Sweden; 9 Expanded Freshwater and Terrestrial Environmental Observation Network (EFTEON), Pietermaritzburg, South Africa; 10 Department of Wildlife, Fish, and Environmental Studies, Swedish University of Agricultural Sciences, 901 83 Umeå, Sweden

**Keywords:** Albedo, Biodiversity, Carbon sequestration, Methane, Natural disturbance, Nature-based solutions

## Abstract

Rangelands face threats from climate and land-use change, including inappropriate climate change mitigation initiatives such as tree planting in grassy ecosystems. The marginalization and impoverishment of rangeland communities and their indigenous knowledge systems, and the loss of biodiversity and ecosystem services, are additional major challenges. To address these issues, we propose the wilder rangelands integrated framework, co-developed by South African and European scientists from diverse disciplines, as an opportunity to address the climate, livelihood, and biodiversity challenges in the world’s rangelands. More specifically, we present a Theory of Change to guide the design, monitoring, and evaluation of wilder rangelands. Through this, we aim to promote rangeland restoration, where local communities collaborate with regional and international actors to co-create new rangeland use models that simultaneously mitigate the impacts of climate change, restore biodiversity, and improve both ecosystem functioning and livelihoods.

## Introduction

Climate change (CC) and biodiversity loss are two of the most urgent and interlinked challenges for the sustainable future of humanity (Arora and Mishra 2021). Whereas CC contributes to biodiversity loss, this loss also drives CC because biodiversity plays a crucial role in cycling of greenhouse gases (GHG) and CC adaptation (Portner et al. 2021; Schmitz et al. 2023). For example, the diversity of terrestrial plants and soil organisms shape two major carbon sinks on land, namely in vegetation and soil (Cavicchioli et al. 2019). Together, these pools are more than twice the size of the atmospheric carbon pool (Friedlingstein et al. 2020) and absorb about 20% of anthropogenic CO_2_ emissions (Le Quéré et al. 2015). After plants and soils, wild animals are increasingly recognized as major drivers of the climate system (Cromsigt et al. 2018; Schmitz et al. 2023). The loss of biodiversity and associated ecosystem functions makes both ecosystems and societies vulnerable to impacts of CC (Mori et al. 2013). Communities that depend directly on the ecosystems they inhabit are particularly vulnerable and often marginalized economically, politically, and geographically (Portner et al. 2021). While such communities frequently contribute the least to CC and biodiversity loss, or to policies that govern them, they tend to be most affected by the impacts of CC (Arora and Mishra 2021). To date, biodiversity conservation and climate action have been insufficient to slow biodiversity loss or CC, and global platforms call for a new paradigm that includes local communities and addresses the dual challenges of the biodiversity and climate crises in an integrated way (Portner et al. 2021).

Addressing biodiversity loss and climate action requires intentional and transformative changes in multiple interconnected systems, including socio-economic, ecological, political, and technological systems (Portner et al. 2021; Smith et al. 2022). Griscom et al. (2017), when discussing natural climate solutions (NCS), recognized part of this inter-connectedness by linking ecological restoration to climate action. They quantified cost-effective ways of increasing carbon storage and reducing GHG emissions while improving soil productivity, cleaning air and water, and maintaining or restoring biodiversity. In fact, Griscom et al. (2017) proposed twenty NCS pathways that protect, manage, and restore ecosystems to mitigate 30% of the GHG emissions target needed to reach net-zero by 2030. Nature-based Solutions (NbS) aims to restore and protect ecological processes and ecosystems to address societal challenges, such as climate change, biodiversity loss and human well-being (Seddon et al. 2020).

Despite debate about the NCS/NbS concept, there is increasing evidence that appropriate NCS/NbS interventions can be beneficial in e.g., reducing exposure to flooding, soil erosion and sea-level rise in coastal communities, climate cooling, and even social inclusion and increasing adaptive capacity to CC through diversifying livelihoods (Seddon et al. 2020). Socio-economic metrics are commonly useful in these examples, but these authors emphasise that metrics need to be context-specific given the complex and varied nature of NCS/NbS interventions. However, the potential of NCS/NbS to deliver positive biodiversity outcomes remains largely untested (Seddon et al. 2020). Girardin et al. (2021) stressed that NbS, as currently implemented, may accelerate biodiversity loss (e.g., when climate action leads to monoculture plantations of non-native tree species) and call for NbS frameworks that are more explicitly inclusive of biodiversity and people. Moreover, several authors argue that NCS/NbS strongly focuses on climate and biodiversity issues but often neglect local societal needs, resulting in counterproductive impacts on local communities (Fischer et al. 2019; Smith et al. 2022). Finally, the NCS and NbS concepts have generated a strong focus on trees, through calls for reforestation and forest conservation. The focus on trees undervalues the climate mitigation potential of non-forest open ecosystems, such as grasslands, savannas and shrublands (Bond et al. 2019). Global large-scale afforestation initiatives (i.e., planting trees in non-forest grassy ecosystems) often directly threaten the biodiversity of these systems and the livelihood of people depending on them (Bond et al. 2019; Fischer et al. 2019). Most of the world’s open ecosystems are used as rangelands to support livelihood through producing meat, fibres and other products. Evidence shows that restoring and sustainably managing rangelands can help mitigate climate impacts through carbon sequestration (Portner et al. 2021). However, the most recent Intergovernmental Panel on Climate Change (IPCC) report on mitigation and adaptation minimally mention of rangelands, except for eating less meat and reducing wildfires (Pathak et al. 2022).

Here, we develop the concept of “wilder rangelands” as an opportunity to restore biodiversity and ecosystem functioning while simultaneously improving human livelihood and mitigating climate impacts by co-creating alternative rangeland uses with local and indigenous peoples. First, we propose an inclusive definition of rangelands and identify problems with current land-use models on rangelands. We define wilder rangelands and outline the mechanisms through which they may help improve human well-being and ameliorate the climate and biodiversity crises. Finally, we explain the implementation of wilder rangelands using a theory of change (ToC) approach outlined by Sullivan and Stewart (2006).

## Inclusive definition of rangelands

Definitions of rangelands vary depending on scientific discipline, education, and management perspectives (Lund 2007). Here, we propose an inclusive definition of rangelands as *extensive rain-fed systems supporting native vegetation that is grazed or browsed by mammalian herbivores that in turn sustain human livelihoods through diverse services, such as the production of meat and fibres or provisioning of diverse recreational activities*. Under this definition, rangelands cover more than 50% of the Earth’s terrestrial surface and include diverse ecosystems, such as grasslands, savannas, shrublands, woodlands, and tundra (ILRI et al. 2021). Rangelands support a diversity of land uses, including livestock and wildlife farmers, pastoralists, hunter-gatherers, tourists, and conservationists (Table 1). Unlike crop farming and agroforestry, rangeland management does not involve homogenizing actions such as clearing, cultivation, biocides, or intensive anthropogenic inputs of seeds and nutrients (Macleod and Brown (2014). Ecosystem services provided by rangelands include cultural (traditional lifestyles, knowledge systems, ecotourism), regulating (carbon uptake and sequestration and animal-plant-soil interactions), supporting (plant primary production), and provisioning (meat, milk, fibre, medicines) services (Costanza 2008). Rangelands thus support billions of people worldwide and are often particularly important for (typically poor) rural communities (Twine 2005).

**Table 1:** Descriptions of the diversity of current land use in rangelands

Land use category	Description
Production of animal products	The most common land use in rangelands ecosystem is the production of wild and domesticated herbivores for their meat, fibres and/or other animal-derived products (e.g., horns as trophies). This land use may take several forms, including private farms and communal grazing areas. They function as either continuous or rotational grazing systems for commercial and subsistence farming. They focus on production of domestic or purely wild herbivores or a mix of them
Harvesting of natural products	People living within and around rangelands depend on rangelands as a source of other, non-animal, products such as firewood, fruits and mushrooms, and medicinal plant materials that are used for direct consumption or to generate income. There are also strong spiritual and cultural values that people associate with rangelands
Recreational use and tourism	Many rangelands function as recreation and tourism areas offering services such as hiking, game drives, horseback riding and hunting for meat or trophies
Conservation	Rangelands support many iconic and endemic species and are also a source of genetic plant and animal materials some of which are in protected areas and used to restore degraded lands. Only 12% of the world’s rangelands are protected within conservation areas in the form of national parks, private game reserves, and private nature reserves
Industrial land uses	Mining activities involving the extraction of a wide array of minerals and fossil fuels are increasingly common in rangelands systems across the world, such as coal mining and fracking in African savannas and grasslands and metal mines in the rangelands of northern Sweden
Emerging land uses	Rangelands, due to their often-open character, are increasingly used for renewable energy production, such as wind and solar farms. Simultaneously, rangelands are also used in carbon offset investments. These investments leverage the natural capacity of rangelands to sequester carbon, thereby contributing significantly to global carbon offset initiatives

## Why do we need to re-think land-use models for rangelands?

The use of rangelands in their present form, for the agricultural purpose of extensive livestock farming, is a crucial threat to wild animal diversity. While livestock and wildlife coexist in the same land, global mammalian biomass is now excessively composed of livestock, comprising about 62% compared to just 2% representing wild land mammals (Bar-On et al. 2018). Threats to wildlife by livestock are largely ancillary via land-use change, removal of predators, and provision of water points, but also direct via disease transmission and persecution (Gordon 2018). Human-caused climate and environmental change increasingly threatens wildlife diversity, while also raising concerns about the sustainability of services provided by rangelands (Havstad et al. 2007). Rangeland faces several critical threats. These include its conversion to other land uses like agricultural intensification, extensive urbanization, mining, afforestation, and invasion by species, as well as the disruption of natural processes like grazing and wildfires. Additionally, climate impacts, such as intensifying droughts, pose a significant threat (Boone et al. 2018; Mashizi and Escobedo 2020). As a result, rangelands experience reduced primary production, greater soil erosion, lower water quality, and loss of ecosystem functions (Bolo et al. 2019). Soil erosion exacerbates climate impacts and lowers nutrient retention, decreasing primary productivity (Bolo et al. 2019). These negative impacts on rangelands affect communities relying on them for livestock production and resource harvesting (Dube et al. 2016).

Some land-use changes partially or completely transform rangelands. Chief among these is crop production, which occupies 10% of habitable land, while urban areas and human infrastructure occupy about 1%, mining (0.01%) and forestry. Throughout the world, changing human population patterns, including urbanization and shifts in rural areas, have complex implications for rangelands. While it is a well-recognized trend that many rural areas in Europe are experiencing depopulation due to urbanization, it is also important to note that this may differ elsewhere. Some regions, for example extensive parts of rural Africa, experienced increased population density in rural areas. This shifting demographic landscape has various impacts on rangelands, including habitat changes associated with increased food production (Ritchie and Roser 2013). With only 12% of global rangelands protected (ILRI et al. 2021), most of these ecosystems are increasingly threatened by such conversions to different land uses, increasing their vulnerability to climate impacts and reducing their ability to provide ecosystem services (Holechek et al. 2020). Accelerating the development of renewable energy infrastructures in rangelands may alter the delivery of crucial ecosystem services provided by rangelands, resulting in adverse ecological impacts on the environment and its users (Kreuter et al. 2016). Renewable energy sources like solar and wind power may decrease GHG emissions but pose trade-offs for biodiversity conservation (Smith et al. 2022), impacting ecosystem services and wildlife, such as bird and bat mortality rates (Agha et al. 2020), disruptions in ungulate migration (Sawyer et al. 2022), and reindeer (*Rangifer tarandus*) avoidance of wind turbines (Skarin et al. 2018).

A more recent threat to rangelands is afforestation funded by carbon credit markets. These markets have gained traction due to growing interest in NCS/NbS and commitments to net-zero emission targets (Tear et al. 2021). Many carbon markets in the agriculture, forestry and other land use sectors have focused on using trees to capture carbon (Bossio et al. 2020). This poses a significant threat to rangelands as afforestation is often promoted in these systems (Bond et al. 2019). The conversion of rangelands to plantations or forests in initiatives such as the African Forest Landscape Restoration Initiative (AFR100) threatens biodiversity, ecosystem services and human livelihood in those non-forest ecosystems (Parr et al. 2014). Soil organic carbon (SOC) and belowground carbon pools in rangelands are also undervalued (Bossio et al. 2020). Despite the importance of fire in maintaining biodiversity in rangelands, recent NCS/NbS discourage burning in savannas and grasslands. However, recent research has shown that fires do not necessarily reduce (Zhou et al. 2022), and may increase (Findlay et al. 2022), SOC in grasslands and savannas.

Current land-use policies and approaches risk undervaluing rangeland ecosystem services. For example, African rangeland policies mainly target livestock production and trade, often neglecting biodiversity as well as crucial social, economic, and environmental aspects needed to sustainably manage natural resources and support pastoralists' livelihoods and access rights (African Union 2013). In many rangelands, communal land usage is customary, allowing pastoralists to move livestock based on forage availability, disease, rainfall, and fire patterns (Behnke and Scoones 1993). However, unregulated grazing and rising livestock numbers on some communal rangelands have contributed to their deterioration (Behnke and Scoones 1993). The shift towards land tenure privatization has disrupted pastoralists' traditional rights and animal movement strategies, frequently without local community input, negatively impacting livelihoods and rangeland management practices (Behnke and Scoones 1993). Without policies that directly address land tenure and communal ownership (African Union 2013), there is high risk that private enterprises (corporate or otherwise), or the establishment of protected areas, replace the use of rangelands by local and indigenous peoples. Yet, indigenous peoples hold essential traditional environmental knowledge including strategies to manage environmental crises and variability such as drought (Selemani et al. 2012; Zhang et al. 2013). Their experience and knowledge could contribute to climate mitigation and biodiversity conservation efforts (Filho et al. 2022). Therefore, co-managing rangelands with local and indigenous peoples is crucial for more equitable and strategic outcomes. Rethinking land-use models for rangelands is crucial to preserve ecosystem services, traditional land rights, and biodiversity, while ensuring environmentally conscious and equitable climate strategies for stakeholders.

## Defining wilder rangelands

**Fig. 1:**
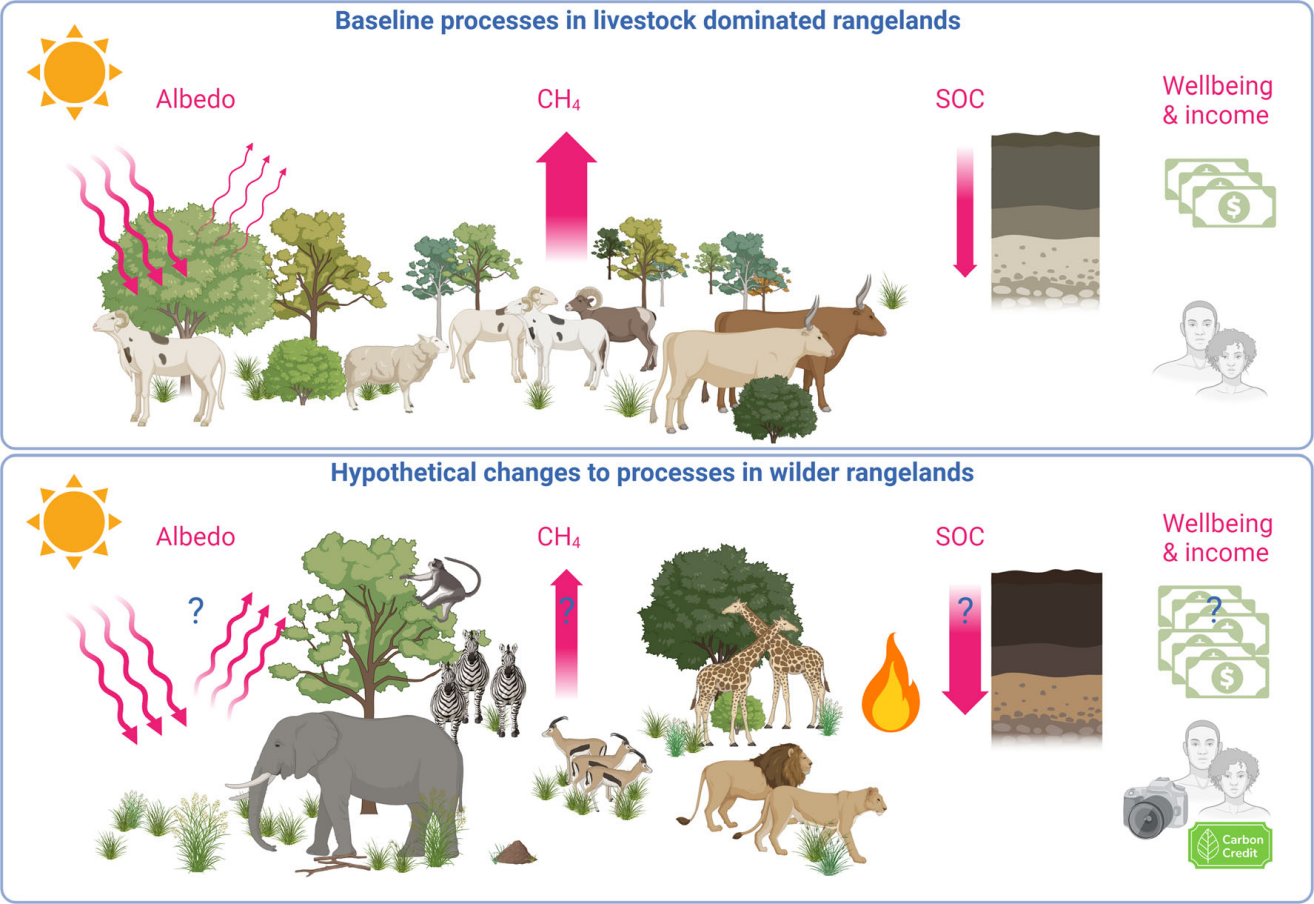
Hypothetical pathways through which wilder rangelands may benefit climate action, biodiversity and livelihoods. Processes such as diverse grazing, browsing, and fire dynamics may result in various ecosystem services such as increased albedo, reduced methane emissions, and increased soil organic carbon (SOC) formation (arrow thickness signifies the magnitude of influence of these services). Other ecosystem services could include habitat provision for wildlife, regulation of fire, erosion control, facilitation of the flow of nutrients, water, and energy, as well as the flows of these services for human wellbeing. Created with BioRender.com

We define wilder rangelands *as open or semi-open ecosystems where natural processes, including diverse grazing, browsing and fire dynamics, prevail. Further, diverse property rights and income models persist. Thus, wilder rangelands provide opportunities to address climate change, halt biodiversity loss, and reduce social inequality,* by restoring biodiversity and providing diverse socio-economic land-use models (Fig. 1). Wilder rangelands are thus biodiverse rangelands, governed by natural processes, where wildlife and livestock may co-exist, while stimulating the indigenous knowledge and use systems that provide livelihoods to local communities. They are resilient to drought and fire, while also storing considerable amounts of SOC (Dass et al. 2018; Findlay et al. 2022) and offer diverse opportunities to sustain livelihoods and the well-being of local communities (Reid et al. 2004).

**Fig. 2:**
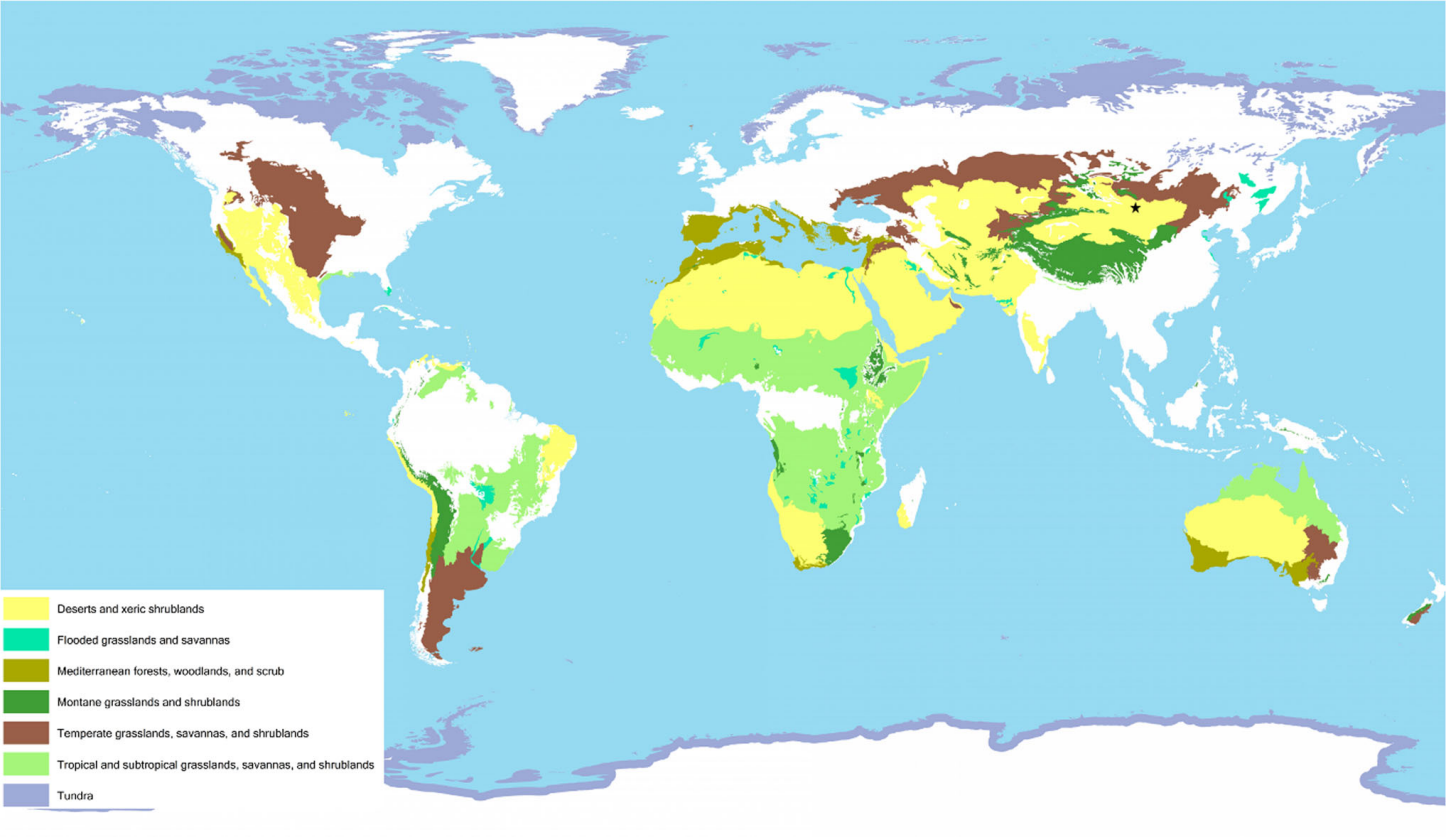
Global Distribution of Rangelands (ILRI et al. 2021) and illustrates the extent of rangelands globally, categorized into seven distinct rangeland types

Diverse rangeland ecosystems across the globe and can be broadly classified into seven major types, each with unique characteristics and ecological significance (Fig. 2). They encompass various landscapes influenced by rainfall, temperature, soil conditions, and management practices (ILRI et al. 2021). These regions provide habitat for various wildlife species unique to these ecosystems. The applicability of the wilder rangelands concept can vary among these different rangeland types and regions. While our case studies provide specific examples, the global map (Fig. 2) allows us to understand the diversity of rangeland ecosystems. The wilder rangelands concept may be more suitable in areas with certain ecological characteristics, and less so in others due to varying levels of native flora and fauna, institutional settings, and environmental conditions. This diversity in rangeland types underscores the need for region-specific strategies and adaptations to the wilding approach.

At the core of the wilder rangelands concept lies the recognition that a diversity of wild animals drives essential natural processes in rangeland ecosystems, such as herbivory, fire, albedo, energy flows, nutrient and water cycles, soil formation and erosion (Table 2), all crucial for the resilience of these ecosystems (Lundberg and Moberg 2003). Herbivory plays a critical role in shaping the structure and composition of vegetation in diverse landscapes with additional benefits for biodiversity, CC mitigation and adaptation. For example, reindeer, moose (*Alces alces*), and other large and small herbivores in the Arctic tundra increase plant species richness by suppressing dominant species (Lindén et al. 2021) and reduce woody cover, resulting in increased surface albedo and a reduction in solar energy absorption at the surface (te Beest et al. 2016), potentially leading to local atmospheric cooling. Wild animals also influence ecosystem resilience through dispersal of seeds and nutrients. While plant species benefit from a diversity of seed dispersers, larger animals play a critical role in the distribution of plant species as their high mobility allows them to disperse seeds over large areas (Correia et al. 2017; Pringle et al. 2023). Similarly, larger animals may be disproportionately important for the redistribution of key nutrients for plants (Doughty et al. 2016; Pringle et al. 2023), and through their physical disturbance and digestive processes may boost the carbon burial in soil (Schmitz et al. 2023). For example, recent work from a Kenyan savanna revealed that SOC and nitrogen stocks decreased with cattle grazing. However, the addition of elephants (*Loxodonta africana*) to the livestock system led to restored SOC stocks (Sitters et al. 2020). Furthermore, many wild mammal species, particularly non-ruminant species such as horses, pigs, rhinos, and elephants, emit less methane than livestock (Jackson et al. 2020). For instance, incorporating kangaroos instead of cattle and sheep in Australian rangelands can cut Australia’s national anthropogenic GHG emissions by 3% annually (Wilson and Edwards 2008). Despite the diverse ways through which wild animals, and their associated natural processes may promote biodiversity restoration and CC mitigation, none of the original twenty NCS pathways defined by Griscom et al. (2017) involved restoring wild animal populations or even diversifying animal feeding guilds (grazers, browsers, megaherbivores) in rangelands or other ecosystems.

**Table 2:** Linking potential wilder rangelands opportunities to rangelands challenges as well as the Sustainable Development Goals and targets

Sustainable Development Goals and targets	Indicators	Rangelands challenges	Wilder rangelands opportunities
Goal 1. End poverty in all its forms everywhere			
1.4 By 2030, ensure that all men and women, in particular the poor and the vulnerable, have equal rights to economic resources, as well as access to basic services, ownership and control over land and other forms of property, inheritance, natural resources, appropriate new technology and financial services, including microfinance	1.4.1 Proportion of population living in households with access to basic services	Overexploitation of natural resources by grazing livestock and other activities, particularly from the of lack of other economic alternatives	New socio-economic land use models, which are more equitable, with a broader portfolio of socio-economic activities, and allow more to profit (e.g., carbon credit programs, renewable energy)
	1.4.2 Proportion of total adult population with secure tenure rights to land, (a) with legally recognized documentation, and (b) who perceive their rights to land as secure, by sex and type of tenure	Change in land tenure systems and access to natural resources and associated income, where communal tenure/use systems are replaced with private ownership, resulting in larger inequality	Wilder rangelands, as a bottom-up approach, provides opportunities for land tenure systems, where individual rights and access are recognized, but embedded in a common use and explicitly linked to social rights and ethics (“modern commons”)
Goal 2. End hunger, achieve food security and improved nutrition and promote sustainable agriculture			
2.4 By 2030, ensure sustainable food production systems and implement resilient agricultural practices that increase productivity and production, that help maintain ecosystems, that strengthen capacity for adaptation to climate change, extreme weather, drought, flooding and other disasters and that progressively improve land and soil quality	2.4.1 Proportion of agricultural area under productive and sustainable agriculture	Poor livestock and/or fire management practices e.g., high stocking rates, inappropriate fire regimes, inappropriate placement of certain livestock or wildlife species in certain ecosystems, lack of spatio-temporal dynamics all of which may negatively affect soils and reduce rangelands productivity	Restoration of natural processes, such as natural grazing and fire regimes, may restore and maintain long-term sustainable production while strengthening capacity for adaptation to climate change, extreme weather, drought flooding and other disasters. Decentralized, and localized, food systems and modern commons may ensure more equitable and fair production and sharing of food
Goal 6. Ensure availability and sustainable management of water and sanitation for all			
6.4 By 2030, substantially increase water-use efficiency across all sectors and ensure sustainable withdrawals and supply of freshwater to address water scarcity and substantially reduce the number of people suffering from water scarcity	6.4.1 Change in water-use efficiency over time	Establishment of invasive species with poor Water Use Efficiency, reducing springs and groundwater resources. Post removal effects of woody invasive plants may include soil erosion and sedimentation of water storage	Eradication of invasive plants and restoration of natural plant cover and processes may reduce erosion /sedimentation of water storage, and increase biotic resistance to new invasions, with biodiversity and water gains
6.b Support and strengthen the participation of local communities in improving water and sanitation management	6.b.1 Proportion of local administrative units with established and operational policies and procedures for participation of local communities in water and sanitation management	Inadequate development and maintenance of water infrastructure in rangelands, e.g., springs. Reliance of people and livestock on the same water resource leads to poor hygiene	Opportunity to engage local communities in water management strategies and policies for improved water infrastructure
Goal 7. Ensure access to affordable, reliable, sustainable and modern energy for all			
7.2 By 2030, increase substantially the share of renewable energy in the global energy mix	7.2.1 Renewable energy share in the total final energy consumption	Air and ground water contamination due to oil and gas exploration and production	Certain renewable energy solutions (solar and wind power) may add to the portfolio of income opportunities
Goal 13. Take urgent action to combat climate change and its impacts			
13.2 Integrate climate change measures into national policies, strategies, and planning	13.2.1 Number of countries with nationally determined contributions, long-term strategies, national adaptation plans and adaptation communications, as reported to the secretariat of the United Nations Framework Convention on Climate Change	Current rangelands practices frequently exacerbate climate change and are poorly adapted to future climates. In addition, rangelands across the world are increasingly presented as opportunities for tree planting and forestation. These tree-based strategies may be inappropriate for the disturbance-prone systems that most rangelands are	Wilder rangelands broaden the portfolio of current climate change mitigation strategies and carry co-benefits for biodiversity and livelihoods. Examples of climate change mitigation opportunities with wilder rangelands include pyric herbivory (i.e., grazing-fire interactions) that leads to cooler fires with more ash stored as carbon in the soil, climate-cooling due to high albedo of grassy surfaces, and reduced methane emissions from mixed wildlife-livestock systems
	13.2.2 Total greenhouse gas emissions per year	Loss of NPP, degradation, soil loss, due to poor grazing practices enhances CO2 emissions and intense livestock practices add to CH4 emissions	Grazing models with either grazing by wildlife or livestock models inspired by how wildlife uses the landscape may enhance soil carbon sequestration and reduce CH_4_ emissions
13.3 Improve education, awareness-raising and human and institutional capacity on climate change mitigation, adaptation, impact reduction and early warning	13.3.1 Extent to which (i) global citizenship education and (ii) education for sustainable development are mainstreamed in (a) national education policies; (b) curricula; (c) teacher education; and (d) student assessment	Lack of awareness of potential for ecosystem restoration, climate mitigation and improving livelihood. Also lack of awareness that tree planting is an inappropriate solution in the many open ecosystems of the world	Opportunity to integrate wild spaces concept into sustainable agriculture curricula and postgraduate studies. Mainstream concept through brochures to public and policy briefs to increase awareness of the opportunities
Goal 15. Protect, restore and promote sustainable use of terrestrial ecosystems, sustainably manage forests, combat desertification, and halt and reverse land degradation and halt biodiversity loss			
15.3 By 2030, combat desertification, restore degraded land and soil, including land affected by desertification, drought, and floods, and strive to achieve a land degradation-neutral world	15.3.1 Proportion of land that is degraded over total land area	Currently rangelands practices frequently lead to soil erosion and desertification and are predicted to do this even more so under climate change	Natural grazing/browsing and fire regimes have the potential to restore lands and maintain vegetation cover to prevent desertification
15.5 Take urgent and significant action to reduce the degradation of natural habitats, halt the loss of biodiversity and, by 2020, protect and prevent the extinction of threatened species	15.5.1 Red List Index	Current rangelands practices are a huge driver of biodiversity loss and increased numbers of endangered wildlife species	Restores natural habitats and processes and also recognizes the huge value of the functional diversity still present in wild herbivore communities and wild fire regimes. This functional diversity provides opportunities for climate change adaptation
15.7 Take urgent action to end poaching and trafficking of protected species of flora and fauna and address both demand and supply of illegal wildlife products	15.7.1 Proportion of traded wildlife that was poached or illicitly trafficked	Poaching	By providing a more diverse income portfolio, available to more people, some of the incentives behind poaching will be reduced
15.8 By 2020, introduce measures to prevent the introduction and significantly reduce the impact of invasive alien species on land and water ecosystems and control or eradicate the priority species	15.8.1 Proportion of countries adopting relevant national legislation and adequately resourcing the prevention or control of invasive alien species	Many current rangelands systems face huge challenges with impacts of invasive alien woody and herbaceous species	Restoration of natural processes may increase biotic resistance to new invasions, e.g., large herbivores and natural fire regimes are being used to reduce re-growth of cleared invasive alien woody plants in South Africa
15.9 By 2020, integrate ecosystem and biodiversity values into national and local planning, development processes, poverty reduction strategies and accounts	15.9.1 (a) Number of countries that have established national targets in accordance with or similar to Aichi Biodiversity Target 2 of the Strategic Plan for Biodiversity 2011–2020 in their national biodiversity strategy and action plans and the progress reported towards these targets; and (b) integration of biodiversity into national accounting and reporting systems, defined as implementation of the System of Environmental-Economic Accounting	Lack of awareness of potential for ecosystem restoration, climate mitigation and improving livelihood. Also lack of awareness that tree planting is an inappropriate solution in the many open ecosystems of the world and such afforestation threatens open ecosystem biodiversity. This reflects a deeply entrenched misunderstanding that grassy ecosystems in areas that can climatically support forest are per definition of anthropogenic origin	Opportunity to integrate wild spaces concept and ecological restoration into agricultural curricula and postgraduate studies. Mainstream concept through brochures to public and policy briefs to increase awareness of the opportunities with wilder rangelands, embracing fire and grazing as key natural processes and not anthropogenic threats

Wilder rangelands offer opportunities to address three critical challenges: CC, biodiversity loss and social inequality, thus addressing the interconnectivity of the United Nations 2030 Agenda Sustainable Development Goals (SDGs) (Table 2). We summarise the current rangeland challenges, how they link to the relevant SDGs, and the envisioned opportunities for wilder rangelands to address these challenges (Table 2). Current mitigation strategies often view biodiversity, climate, and society as separate, overlooking their interactions (Pascual et al. 2022). For example, agricultural production is a common intervention to fight poverty but can harm biodiversity and climate by replacing diverse ecosystems with monoculture livestock or crops (Fleischner 1994). This may result in increased GHG emissions due to the loss of critical ecological functions, such as carbon uptake and sequestration, essential for maintaining ecosystem balance (Poore and Nemecek 2018).

Moreover, current CC mitigation strategies often focus on monoculture tree plantations sensitive to global changes (Hulvey et al. 2013). They are often wrongly suggested as afforestation of open ecosystems that are more resilient than plantations and offer greater and longer-term carbon storage (Seddon et al. 2019). These strategies demand vast land areas, causing declines in agricultural land and biodiversity, while exacerbating negative impacts on local communities relying on resources and biodiversity (Doelman et al. 2020). For example, a program aimed to increase carbon sequestration in the Brazilian tropical savanna through afforestation and fire suppression led to a decline in plant and ant diversity (Abreu et al. 2017). Similarly, a program in Uganda planted "climate forests"—as monocultures of alien pines—on communal rangelands, destroying access to valuable trees and grazing land for local communities (Fischer et al. 2019).

## Implementation of wilder rangelands

**Fig. 3:**
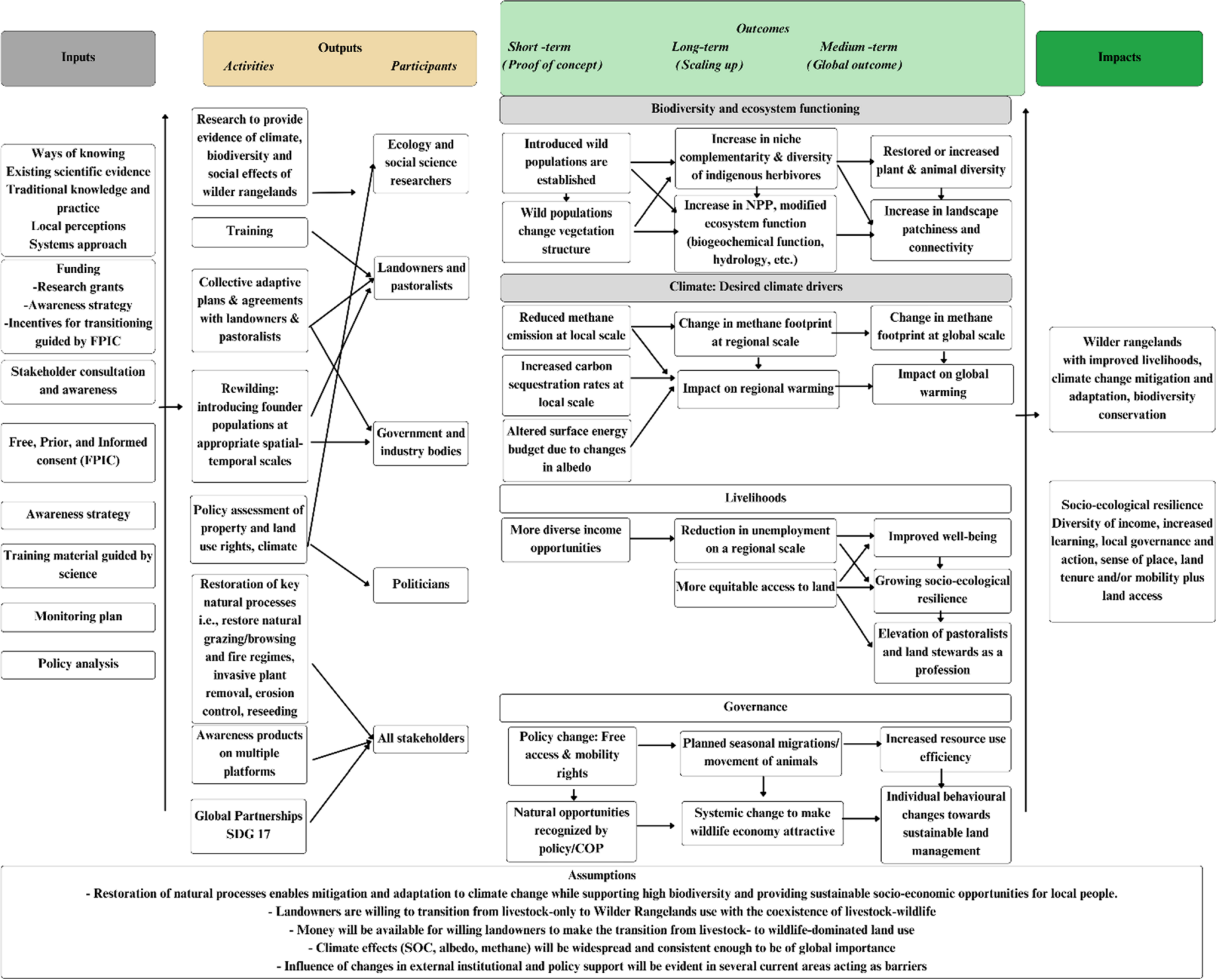
This diagram illustrates the interconnected elements in the Logic model for the wilder rangelands Theory of Change, including inputs, activities, participants, outcomes, impacts and assumptions underpinning the transition from livestock-dominated rangelands to 'wilder rangelands

We followed a ToC approach for developing and implementing wilder rangelands, as it is an efficient tool for designing, monitoring, and evaluating complex, multifaceted and long-term initiatives (Sullivan and Stewart 2006). This approach explores causal linkages between desired changes expected from certain interventions at different result levels (activities, outputs, outcomes, and impact), the actors, and factors influencing those changes (Fig. 3). Important activities needed to implement wilder rangelands are highlighted (Fig. 3). The illustrated ToC is linear for the sake of simplicity and readability, but we acknowledge that unexpected and complex interactions may occur as a result of wilder rangelands.

### Restoration of key natural processes

Restoring natural processes under the proposed wilder rangelands framework can entail fully or partially replacing managed livestock systems with communities of native wild or semi-domesticated native herbivores, with or without regulated hunting. Wilder rangelands can also include actively restoring or maintaining the traditional and extensive forms of livestock grazing, such as reindeer husbandry or traditional African pastoral systems, that share many features with wild grazing systems. Such traditional systems have allowed human populations to settle and persist for many thousands of years in close connection with the landscape, maintaining the landscape’s biodiversity and ecological functioning (Stoessel et al. 2022). Re-introduction of wild or semi-domesticated native herbivore regimes, or traditional pastoral systems, serves to restore important interactions between ecosystems and mammalian herbivores (i.e., herbivory, pollination, seed and nutrient dispersal, herbivore-fire interactions) that are essential for ecosystem functioning (Holtmeier 2015). Studies show that many plant species can resprout following herbivory by native mammals and insects (Midgley 1991) or have evolved defence mechanisms (i.e., chemical defence) against different types of herbivories (Leimu et al. 2012). A good example is the ability of *Portulacaria afra* to regenerate from discarded branches after browsing by elephants (Stuart-Hill 1992). Another example is the circumpolar browsing of *Betula nana* by reindeer and caribou, where multiple chemical defence strategies have evolved in the same species complex (Lindén et al. 2022).

Wild herbivores and frugivores are essential in seed dispersal and in promoting germination (Fazelian et al. 2014). For instance, elephants help *Sclerocarya birrea* seeds germinate by cracking shells while chewing, increasing germination rates (Midgley et al. 2012). Wild herbivores are also central to nutrient cycling, e.g., reindeer elevate nutrient cycling, raising plant nitrogen levels and primary production (Olofsson et al. 2004). Herbivory by multiple species also plays a crucial role in regulating the frequency and distribution of fire (Fuhlendorf et al. 2009; Cromsigt et al. 2018) and creating habitat heterogeneity (Fuhlendorf et al. 2009). For example, diverse tundra herbivores preserve grass, moss, and shrub mosaics by limiting shrub and tree densities via trampling and browsing (Olofsson and Post 2018). In this way, reindeer slow down ecosystem responses to CC, e.g., by mitigating warming effects on the tundra, carbon balance or increasing summer albedo (Väisänen et al. 2014; te Beest et al. 2016).

Rewilding with native herbivores may play a role in restoring degraded rangelands. For example, restoring native ungulates in Gorongosa National Park in Mozambique led to a decline in invasive plant species (Guyton et al. 2020). However, rewilding significantly degraded rangelands may also reduce the ability of vegetation to regenerate (Kerley et al. 1995), thus resulting in further and irreversible negative impacts on rangelands. Therefore, as a first step before rewilding native herbivores, it is essential to restore significantly degraded rangelands to a state that can support the intended wildlife or livestock. Restoring degraded rangelands can involve planting native (herbaceous) species, eradicating invasive species, or allowing a grazing rest period for the natural regeneration of plant species (Genes and Dirzo 2022).

Restoring natural processes may entail ‘rewilding’ livestock grazing practices through learning from wild grazing or traditional herding systems or allowing for mixed livestock-wildlife systems. The rapid human population growth has increased the need to expand agricultural lands (Fróna et al. 2019) compromising biodiversity and ecosystem functioning. However, appropriate livestock management practices such as low stocking rates and rotational grazing can enhance rangeland heterogeneity, offering greater foraging options for livestock and wild herbivores (Fynn et al. 2016; Cipriotti et al. 2019). Venter et al. (2017) suggests mimicking migrations in wild grazing systems by adjusting stocking rates to suit the dry and wet seasons and incorporating return periods. However, return periods and stocking rates depend on several local factors, such as rainfall, soil types and vegetation (Fynn and O’Connor 2000), of which our knowledge is currently limited. Rangelands and their plant communities have co-evolved with wild herbivores, and further studies are required to understand and effectively mimic wild grazing patterns.

Additionally, retaining or restoring the diversity of herbivore species with different feeding habits (e.g., grazing and browsing herbivores of different sizes) can effectively use the vegetation and diversify ways to generate income (Abate et al. 2010). Another opportunity lies in the judicious use of temporary enclosures (“kraals” or “bomas”) from traditional livestock systems, which not only mitigate human-wildlife conflict but may also create nutrient and carbon hotspots (Momberg et al. 2023) through concentrated deposition of dung and urine (Fynn et al. 2016). The application of kraaling should be context dependent. Hawkins et al. (2022) found that in South African mesic grasslands, kraaling increased grass basal cover when the initial cover was low. However, it decreased basal cover when the initial grass and herbaceous plants cover were relatively high. On the other hand, in savanna sites across southern Africa, kraaling consistently increased basal cover, as observed by Momberg et al. (2023).

Privatisation and fencing of pastoral lands and fencing protected areas, reduces the ability of wild herbivores and livestock to move in response to spatial and temporal variability in resources and rainfall (Western et al. 2009; Fynn and Bonyongo 2011). Within the proposed wilder rangelands, we foresee opportunities for grazing practices that allow for coexistence of livestock and wild herbivores that will benefit both livestock production and biodiversity conservation and allow herbivores to roam freely across larger landscapes (Fynn et al. 2016), e.g., by recognising the importance of traditional migratory path or to (re-) establish migration or movement corridors. These opportunities include the restoration of animal movements, which allows both the migration of herbivores and the redistribution of nutrients. Increased herbivore diversity and functional heterogeneity may positively influence vegetation structure (Fynn et al. 2016) and enable diverse income streams, such as restoring pastoralist routes (see below). For thousands of years pastoralists managed their domestic animals amongst wild herbivores following similar seasonal movement patterns (Fynn et al. 2015), but increasingly their movements have been obstructed. The mobility of large wild and domestic herbivores generates landscape-level heterogeneity through herbivory, trampling and nutrient cycling resulting in greater productivity and sustainability of livestock and wild herbivores (Owen-Smith 2004). Introducing wild herbivores into livestock grazing systems may aid restoration of degraded rangelands while also mitigating CC. For example, Wells et al. (2022) found that reducing cattle densities coupled with the presence of megaherbivores increased cattle foraging efficiency while benefiting mesoherbivores, such as zebra (*Equus* sp).

### Addressing land-tenure issues

Addressing land-tenure systems is paramount in a world where land resources are becoming increasingly scarce and contested. This is particularly crucial for wilder rangelands, as flexible and inclusive land tenure management will enable land connectivity, wildlife movement and transhumance, thus fostering healthier ecosystems that can better withstand CC impacts. Due to increased pressure and competition for natural resources, governments and investors have become increasingly interested in rangelands—areas previously often deemed as marginal lands (Robinson and Flintan 2022). These common lands are often treated as “*terra nullius*” (nobody’s land) by governments and investors (Fischer et al. 2019), despite having been inhabited and used by people, including pastoralists, for thousands of years. Consequently, these lands lack protection and secured land tenure, making them prone to individualization and privatization with cumulative pressures from various forms of land use (Stoessel et al. 2022). As highlighted by Doss and Meinzen-Dick (2020), if we want to maintain these types of rangelands, there is a need for legal and secure land tenure. Importantly, these land-tenure models must anticipate and address conflicts resulting from long-standing parallel land uses (Sandström et al. 2006). For example, in the Arctic region, the expansion of mining and forestry conflicts with the use of rangelands by Sámi reindeer herders. Their traditional knowledge is seen as a secondary source of information in discussions around the use of arctic rangelands. Here, it is essential to integrate human rights impact assessments into existing license-granting mechanisms and examine companies' compliance with applicable human rights norms for local and indigenous populations (Petrétei 2020). Thus, it is crucial to ensure proper consideration of the special status and interests of the Sámi people and similar indigenous groups elsewhere during the planning and implementation of extractive industrial projects to guarantee fair treatment of all stakeholders involved.

Another example that could inform wilder rangelands is Natura 2000, a 30-year-old Europe-wide network of nature reserves, voluntary land conservation agreements, abandoned land, and private land that recognizes pastoralists and enables the cultural practice of transhumance. This policy recognizes the potential benefits of shepherding for biodiversity through e.g., nutrient redistribution, seed dispersal, control of woody encroachment, maintenance of landscape heterogeneity, wildfire control, and supply of livestock carcasses in various open habitats such as natural and semi-natural grasslands, wooded pastures and meadows, scrub, and heathlands (European Union 2018). Current initiatives to support these practices include incentive payments, markets for products of origin (e.g., cheese) and shepherding schools. Finally, wilder rangelands could be modelled on certain traditional communal rangeland systems, e.g., pastoralists in the Herding for Health Programme (Heermans et al. 2021) in Mozambique have access to their communal areas as well as private land and nature reserves to allow transhumance (Anna Jean Haw, pers comm, 2023). These examples serve as strong lessons for other parts of the world, where the use of rangelands by indigenous communities’ conflicts with other stakeholders.

Policymakers must work with ecological and social scientists to develop and implement policies that encourage land uses that maintain natural ecosystems and enhance functional resilience, thus uniting biodiversity, and climate impacts.

### Inclusion of local communities belonging to rangelands

Local communities have often been displaced or excluded from areas previously used for grazing and harvesting natural products, resulting in resistance against conservation initiatives (Fynn et al. 2016) and afforestation efforts (Fischer et al. 2019). More recently, this problem is acknowledged and different, more inclusive models, are suggested in conservation. For example, the premise of transfrontier conservation areas (TFCAs) in southern Africa was to explicitly include the socio-economic development of rural communities via ecotourism, alongside global biodiversity and regional integration goals. However, cultural values and land-use needs such as crop and livestock farming were, and still are, often forgotten in this process. For example, due to increased human-wildlife conflict, coexistence with wildlife was costlier than anticipated for people living on the edge of TFCAs in Zimbabwe, South Africa and Mozambique (Andersson et al. 2017). This makes it imperative that governance systems must include all stakeholders in the planning and execution of global crisis interventions, especially local communities whose goals, values and behaviours determine the success of these interventions (Pascual et al. 2022). Importantly, people in local communities in these rangelands have knowledge that could benefit our understanding of local conditions, as well as strategies to adapt to changes in biodiversity and climate (IPBES et al. 2019). Wilder rangelands specifically recognise that solutions to global challenges should be supported by, and ideally co-created with, the communities living in the rangelands, by recognising that ecosystems reflect many ecological processes, including those of indigenous peoples (Ogar et al. 2020), for the restoration of degraded landscapes (Reyes-García et al. 2019). The wilder rangelands concept incorporates traditional or indigenous knowledge systems that have evolved through livelihoods dependent on these ecosystems.

### Creating diverse income opportunities

Rangeland degradation, disease, and transportation issues hinder small farmers from accessing markets and generating income (Flintan et al. 2011; Beyene et al. 2014). Wilder rangelands may offer diverse land-use opportunities including adaptive wildlife-livestock grazing, conservation, tourism, hunting, and renewable energy (Macleod and Brown 2014; Reid et al. 2014). Certain initiatives take a One Health approach, acknowledging the interconnection between human, animal, and environmental health, e.g., the southern African Herding for Health programme (Heermans et al. 2021). The latter programme trains farmers in rangeland management, animal husbandry, resolution of human-wildlife conflict, and facilitates access to profitable markets. Non-traditional livestock species offer additional revenue options from meat, milk, and hair/fibre production. For example, Bactrian (*Camelus bactrianus*) and dromedary (*Camelus dromedarius*) camels, llama (Lama glama), alpaca (*Vicugna pacos*), guanaco (*Lama guanicoe*), and vicuña (*Vicugna vicugna*) are becoming increasingly attractive as livestock due to their ability to adapt to harsh climates and high altitudes with income opportunities from their meat, milk, and hair/fibre production (Zarrin et al. 2020). Integrating renewable energy infrastructures such as solar and wind power into rangeland is an opportunity to diversify revenue streams while facilitating climate action. Landowners can generate clean, renewable energy without compromising the grazing capacity of mammalian herbivores by installing solar panels and wind turbines in less productive areas of the rangeland. They may further benefit by leasing land for renewable energy infrastructures while allowing grazing or other traditional rangeland uses (Ott et al. 2021). However, an integrative assessment of energy development on rangelands is necessary to minimise environmental impacts associated with different types of energy developments while balancing traditional rangeland uses.

Incentives to restore rangeland biodiversity and decrease GHG are also potential income sources. These opportunities include a subset of the diversity of carbon and biodiversity credit programs that are currently prevalent and increasing. Carbon sequestration through initiatives such as vegetation restoration and planned grazing can generate revenue while conserving the environment (Tennigkeit and Wilkes 2008). For instance, transitioning from late dry-season severe fires to early dry-season cooler burns may yield carbon credit revenues due to reduced CO_2_ emissions and increased carbon sequestration in soil and above-ground biomass (Tear et al. 2021). Central to such opportunities would be to ensure they do not disrupt social structures or indigenous practices. Fire and grazing management practices can also benefit local communities through employment or costs associated with implementing these programs. In addition, local communities may also benefit from larger quantities and higher quality forage for their livestock, improved nutrient cycling, increased water storage capacity, and a reduction in tick-borne diseases, all of which result from altered grazing and fire management practices (Odadi et al. 2017; Tear et al. 2021). Developing a biodiversity credit market system for rangelands is still in its infancy. Certain standards explicitly include carbon, biodiversity and communities but have been criticised for being market-orientated and neglecting participation or poverty alleviation (Melo et al. 2014).

### Navigating the Socio-Political and Institutional Landscape of Rangelands

The successful implementation of wilder rangelands for restoring biodiversity, enhancing ecosystem functioning, improving human livelihoods, and mitigating climate change necessitates a comprehensive examination of the socio-political and institutional factors that can enable or hinder these objectives (Behnke 2021). This section delves into the intricate web of socio-political, governance, and institutional elements that shape wilder rangelands and emphasise the need for an integrated perspective.

#### External socio-political-economic interests and their implications

Throughout the preceding sections, we have touched upon various policies and associated socio-economic interests that play a pivotal role and currently often act as barriers to the concept of wilder rangelands. These policies encompass mining, forestry, energy developments, but also conservation, and carbon and biodiversity credit schemes. It is evident that while some of these policies are essential for the successful implementation of wilder rangelands, others, despite good intentions, may inadvertently become barriers to progress (Behnke 2021). Hence, to identify potential barriers to wilder rangelands it is necessary to conduct a policy analysis to identify policies that may support or may hinder implementation and identify what type of policy instruments (regulatory, economic or informative), or mixed policy instruments, could incentivise a change in the direction of wilder rangelands. However, it is also necessary to conduct an institutional analysis to identify current institutional factors such as land tenure, existing governance systems, and their implications for a wilder rangelands perspective. Here it is necessary to address rights-based perspectives to avoid the risk of violating human rights. Behnke (2021), who provides a 'realistic approach to politics' perspective, also highlights the need to recognize irreversible changes in pastoral systems. He underscores the role of modern capitalist institutions in shaping rangelands and offers insights into principles and lessons of traditional approaches that can be updated to meet new realities. For instance, East African pastoralists have adapted to changing environmental conditions, including climate change, by diversifying their herds to include more browsers like camels and goats. This shift in herd composition is a direct response to the threat of grass scarcity, demonstrating the flexibility of traditional pastoral approaches in the face of new realities.

Moreover, migratory rangeland systems offer a distinct approach in managing livestock, contrasting with fenced rotational systems. The former capitalizes on environmental heterogeneity, utilizing the varying landscape and seasonal changes to benefit both livestock and biodiversity. This can be seen in how pastoralists have amplified rangeland heterogeneity for millennia. On the other hand, fenced ranching often attempts to suppress heterogeneity, focusing on uniformity of grazing distribution. The pressures from market penetration and state policies have led to shifts in indigenous tenure regimes, often resulting in rangeland fragmentation and reduced mobility for pastoralists. However, the emergence of novel land tenure arrangements, such as conservation easements and grass banking in American ranching, suggest potential strategies for preserving the open-range migratory systems, aligning with traditional approaches.

#### An integrated perspective

Adopting an integrated perspective is crucial to tackle the diverse policy and institutional challenges. This approach should consider the complex interplay of socio-political factors, governance, and institutional dynamics, as emphasized by Behnke (2021). Additionally, Brunson et al. (2022) advocate for intergrating social sciences into the traditionally natural science-focused rangelands research and emphasize the need for social justice perspectives in the policy agenda. They encourage considering a broader range of knowledge sources and stress the importance of listening to diverse voices in rangeland management. Here, we highlight that our proposals also strongly align with the growing international recognition and call the listening to, and acting upon, local and indigenous knowledge and practices. Strengthening the wilder rangelands can be achieved by connecting with initiatives focusing on indigenous and local knowledge and practices. Key initiatives include the IPBES, IPCC, and the Kunming-Montreal Global Biodiversity Framework. The integrated approach we advocate will enrich our understanding of the factors influencing policy directions and governance (Nori and Scoones 2023). Nori and Scoones (2023) also highlight the importance of addressing local communities' welfare in policy objectives and challenging bureaucratic approaches and distorted interests. While many policy issues are externally driven, it is necessary to acknowledge the presence of local socio-political conflicts. These conflicts can stem from differing interests between local elites and more traditional local or indigenous groups. Nori and Scoones (2023) note that even when elite pastoralists are co-opted into state structures, pastoralist populations may feel left behind by ongoing transformations. Addressing these conflicts is essential to ensure that the implementation of wilder rangelands benefits all stakeholders.

#### Changing the rangeland degradation narrative

One deep-lying barrier to the idea of wilder rangelands is the widespread paradigm and narrative that many of the world’s rangelands, and grassy biomes they are a part of, are of anthropogenic origin (Parr et al. 2014). According to this paradigm, anthropogenic fire and/or grazing regimes have led to deforestation and created these grassy systems under climatic conditions that would naturally allow forest (Veldman 2016). According to this view, grassy ecosystems are considered degraded forests, except for places that are too cold or dry for trees (Veldman 2016). This paradigm forms a barrier to wilder rangelands, because it sees fire and potentially even grazing as “unnatural” anthropogenic processes. Consequently, the restoration of rangelands towards more natural ecological states would depend on excluding fire and/or grazing. This “degradation narrative” is used increasingly to advocate for carbon forestry and afforestation, i.e., tree planting in grassy ecosystems (Hajdu et al. 2016; Hajdu and Fischer 2017; Bond et al. 2019). There is now ample evidence, however, that grassy biomes and their fire and grazing regimes are not of anthropogenic origin but are ancient systems, even under climatic conditions that are wet and warm enough to support forest (Veldman et al. 2015; Bond and Zaloumis 2016). Fostering a more widespread acceptance of this alternative paradigm would be an essential enabling factor accepting and implementing of wilder rangelands.

## Conclusions

This paper explores wilder rangelands as a solution for restoring biodiversity, supporting human livelihood, and mitigating CC. Restoring natural processes, such as herbivory, animal movement, and fire regimes, is crucial for effective climate action. Wilder rangelands integrate scientific and indigenous knowledge to create sustainable strategies that protect ecosystems and local communities. Key actions involve respecting indigenous knowledge systems, community engagement, secure land tenure systems for communal use, and alternative income sources. These strategies promote holistic climate solutions that improve local community livelihoods. Supported by traditional grazing systems, wilder rangelands integrate diverse land uses like animal production, tourism, carbon credit markets, and renewable energy development to mitigate CC while enhancing biodiversity. Given the potential trade-offs among different land-use strategies in wilder rangelands, further research is needed. Ensuring robust environmental assessments and collaborative decision-making is paramount for positive ecological and social outcomes. Successful implementation of wilder rangelands requires collaboration among local stakeholders, scientists, policymakers, and communities to develop appropriate protective policies.
